# Chemical Synthesis of La_0.75_Sr_0.25_CrO_3_ Thin Films for *p*-Type Transparent Conducting Electrodes

**DOI:** 10.1021/acs.chemmater.2c03831

**Published:** 2023-04-13

**Authors:** Pamela Machado, Roger Guzmán, Ramon J. Morera, Jordi Alcalà, Anna Palau, Wu Zhou, Mariona Coll

**Affiliations:** † Institut de Ciència de Materials de Barcelona ICMAB-CSIC, Campus UAB, Bellaterra 08193, Spain; ‡ School of Physical Sciences, University of Chinese Academy of Sciences, Beijing 100049, China

## Abstract

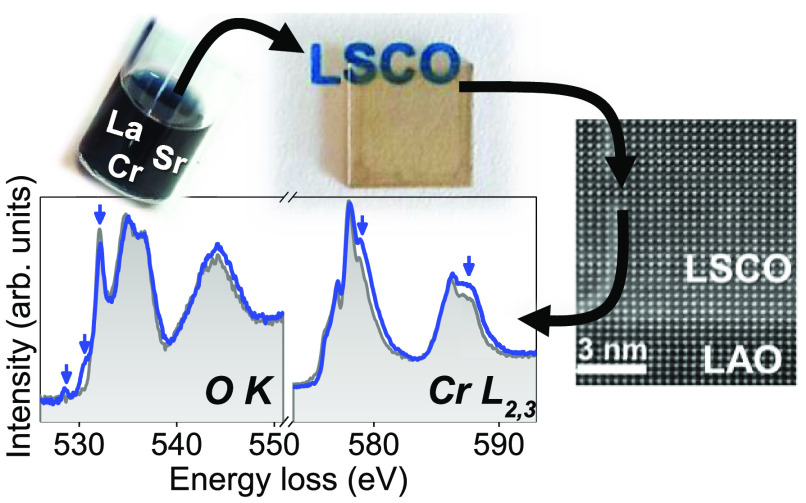

The imperative need for highly performant and stable *p*-type transparent electrodes based on abundant metals is stimulating the research on perovskite oxide thin films. Moreover, exploring the preparation of these materials with the use of cost-efficient and scalable solution-based techniques is a promising approach to extract their full potential. Herein, we present the design of a chemical route, based on metal nitrate precursors, for the preparation of pure phase La_0.75_Sr_0.25_CrO_3_ (LSCO) thin films to be used as a *p*-type transparent conductive electrode. Different solution chemistries have been evaluated to ultimately obtain dense, epitaxial, and almost relaxed LSCO films. Optical characterization of the optimized LSCO films reveals promising high transparency with ∼67% transmittance while room temperature resistivity values are 1.4 Ω·cm. It is suggested that the presence of structural defects, i.e., antiphase boundaries and misfit dislocations, affects the electrical behavior of LSCO films. Monochromated electron energy loss spectroscopy allowed changes in the electronic structure in LSCO films to be determined, revealing the creation of Cr^4+^ and unoccupied states at the O 2*p* upon Sr-doping. This work offers a new venue to prepare and further investigate cost-effective functional perovskite oxides with potential to be used as *p*-type transparent conducting electrodes and be easily integrated in many oxide heterostructures.

## Introduction

Transparent conducting oxides (TCOs) constitute a special class of materials that combine properties of high electrical conductivity (σ > 10^4^ S·cm^–1^) and high optical transparency in the visible spectrum (*T* > 80%) with use in touch screens, solar cells, and smart windows.^1−7^ From the last 50 years, *n*-type Sn:In_2_O_3_ (ITO) thin films have been the most utilized TCO because of their excellent balance between transparency and electrical conductivity, but their high and fluctuating price as a consequence of indium scarcity demands for the study of alternative candidates. Among the enormous variety of oxides, the interest in the family of perovskite oxides for TCOs arises from the wide range of available compositions and from their structural compatibility with many functional complex oxides, making them useful to prepare innovative and stable heteroepitaxial devices for electronic and energy applications.^8−12^ Indeed, CaVO_3_, SrVO_3_, and SrNbO_3_ are promising *n*-type TCOs that have been demonstrated to show excellent performances when compared with ITO.^13−15^ To broaden the applications of TCOs and continue advancing functional perovskite-based heterostructures, there is a strong need to find high performance *p*-type TCO counterparts, although their development is intrinsically challenging. The localized nature of O 2*p* orbitals at the top of the valence band of most metal oxides hinders the introduction of shallow acceptors and large hole effective masses.^16−18^ Chemical modulation of the valence band, which consists of introducing covalency in the metal–oxygen bond, can overcome this issue.^19^ Consequently, closed and quasi-closed shell electronic configurations of cation species (*d*^10^, *d*^6^, *d*^3^) are the best candidates to reduce localization of holes in oxygen ions and minimize colorization from the metal d–d excitation. In this line, some new *p*-type TCOs based on Cr^3+^ oxides (*d*^3^ in octahedral configuration) have been already identified^20−22^ among which Sr-doped LaCrO_3_ appears as a strong candidate with reasonably high transparency and *p*-type conductivity.^23−26^ Importantly, the ability to grow these materials as epitaxial films can be used as a lever to tune the physical properties.^27−29^ In fact, it has been recently demonstrated that electric conductivity of Sr-doped LaCrO_3_ films grown by molecular beam epitaxy (MBE) can be finely tuned through epitaxial strain.^26^ Nevertheless, when evaluating the suitability of an oxide material to be used as a TCO, along with high electrical conductivity and optical transmittance, it is important to consider cost-effective and sustainable processing techniques. Over the past decade, significant progress has been made to enable the production of high quality metal oxide thin films using chemical solution deposition (CSD), a well-established, inexpensive, and potentially scalable route that offers the possibility to prepare epitaxial perovskite oxides and perform simple compositional tuning by identifying compatible precursor salts and solvents.^30−32^ In fact, successful growth of solution-processed *n*-type TCO perovskite films based on M-doped BaSnO_3_ (M = La, Pr, Nd, Sb)^33−36^ and La-doped SrTiO_3_^37,38^ has been previously reported. However, the study of CSD *p*-type TCO perovskite films is at its infancy. Thus, despite the promising perspectives of this approach, the syntheses of many new and attractive complex oxides films, including Sr-doped LaCrO_3_, remain unexplored.

Motivated by the design of an nonexistent cheap chemical route to fabricate *p*-type Sr-doped LaCrO_3_ epitaxial films, in this work we explore the use of CSD through a systematic study of the influence of solution chemistry on gel thermal decomposition and film morphology by means of thermogravimetric analysis, viscosity, and scanning electron microscopy. Detailed structural analysis has been performed by X-ray diffraction and scanning transmission electron microscopy. Film optical transparency and electrical resistivity have been evaluated on the optimized 45 nm films of La_0.75_Sr_0.25_CrO_3_ (LSCO) by spectroscopic ellipsometry and temperature dependent resistivity measurements, respectively. Monochromated electron energy loss spectroscopy allowed the electronic properties of these solution processed oxides to be unraveled.

## Experimental Section

### Preparation of LSCO Thin Films

#### Solution Preparation

Stoichiometric amounts of anhydrous strontium nitrate, Sr(NO_3_)_2_, hydrated lanthanum nitrate, La(NO_3_)_3_·6H_2_O (99.999%), and chromium nitrate, Cr(NO_3_)_3_·9H_2_O (99.9%), from Sigma-Aldrich, were weighted and mixed to prepare four 0.25 M solutions by modifying the solvent (2-methoxyethanol, MOE, acetic acid, AcA, and water) and additive composition (*N*,*N*-dimethylformamide, DMF, acetylacetone, AA, diethanolamine, DEA, and citric acid, CA); see Table 1. According to their chemical formulations, the four solutions investigated are named MOE, DMF-AA, DEA, and CA. For the preparation of the solutions denoted MOE, DMF-AA, and DEA, the metal nitrate precursors were mixed in an organic solvent blend of 2-methoxyethanol and acetic acid (3:1). DMF-AA solution was obtained from the addition of *N*,*N*-dimethylformamide and acetylacetone as stabilizers in a molar ratio to total metal cations of 1:2, whereas DEA solution was prepared from the addition of the chelating agent diethanolamine in a molar ratio to Cr of 1:4. On the other hand, CA solution was obtained from mixing the nitrate precursors in water and using citric acid in a molar ratio of total metal cations of 2:1 to minimize the hydrolysis and condensation reactions. The viscosities of all 0.25 M LSCO precursor solutions were measured at 22 °C at a fixed speed of 2880 s^–1^ for 30 s using a Haake RheoStress RS600 rheometer from Thermo Electron Corporation.

**Table 1:**
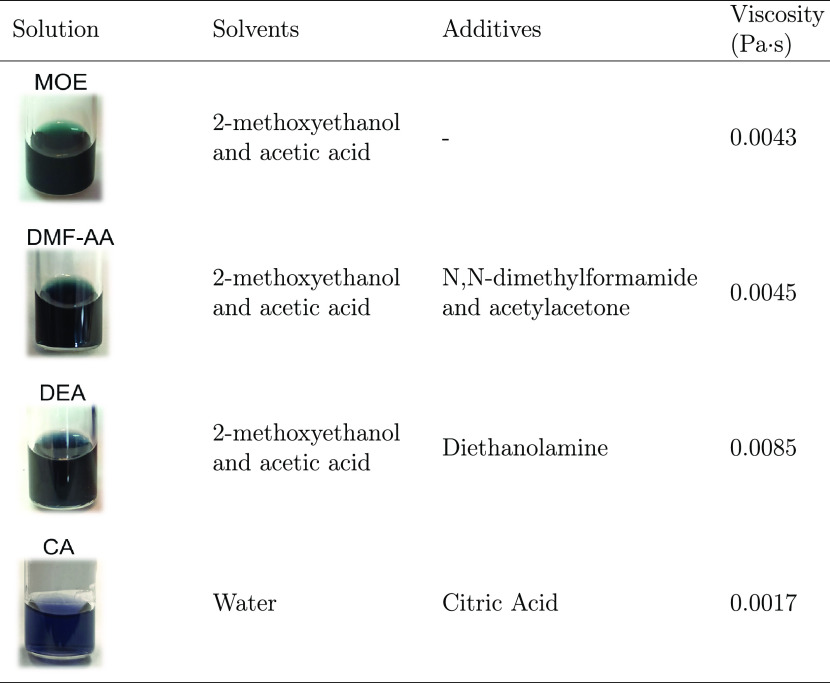
Description of the Four LSCO Precursor Solutions’ Compositions (Solvent and Additive) and Viscosities

#### Thermal Decomposition Characterization

Simultaneous thermogravimetric analysis (TGA) and differential scanning calorimetry (DSC) were carried out with a thermal analyzer TGA/STA 449 F5 Jupiter from NETZSCH at University of Alicante, Spain, with a range of temperatures from 30 to 900 °C and a heating rate of 30 °C·min^–1^ under an O_2_-rich atmosphere, i.e., N_2_:O_2_ (1:9), for MOE, DMF-AA, DEA, and CA solutions, and air atmosphere, i.e., N_2_:O_2_ (4:1), for DEA solution. Both atmospheres used a continuous flow of 10 mL·min^–1^. In order to perform this study, the precursor solutions were previously dried to a gel by an R-210/215 rotary evaporator from Büchi.

#### Thin Film Preparation

LSCO thin films were prepared by means of CSD on two different monocrystalline substrates: (00l)-SrTiO_3_ (STO) and (00l)-LaAlO_3_ (LAO). For film growth optimization and optical transmittance measurements, films were prepared on STO, whereas for electrical conductivity and advanced structural and electronic analysis, films were grown on LAO. For specific comparisons, the LaCrO_3_ (LCO) parent compound thin films have also been prepared. A total of 18 μL of L(S)CO precursor solution was spin-coated on STO and LAO at 6000 rpm for 36 s in a N_2_ box with relative humidity < 20%. The spin coated samples were exposed to a low-temperature treatment at 100–400 °C for 4–30 min. Subsequently, samples were introduced in a preheated tubular furnace at 850 °C in air or under continuous O_2_ flow of 0.6 L·min^–1^ for 45 min and quenched to room temperature. This procedure leads to film thicknesses of 40–70 nm for the different solution chemistries.

### Thin Film Characterization

#### Surface Morphology

Surface morphology images were acquired by scanning electron microscopy (SEM) using a QUANTA FEI 200 FEG-ESEM scanning electron microscope equipped with energy dispersive X-ray spectroscopy (EDX). EDX analysis was performed at 10 kV for 70 s. Surface morphology and root-mean-square (rms) roughness were studied from the topography images acquired with an atomic force microscopy (AFM) Keysight 5100 instrument in dynamic mode and analyzed by MountainsMap Premium 9 software from Digital Surf.

#### Optical Characterization

Optical transmittance (%T) of L(S)CO thin films on STO was studied in the wavelength range of 300–1000 nm with a spectroscopic ellipsometer SOPRALAB GES5E in photometry mode, which uses a Xe lamp as a light source. The light source was incident in the film side with an angle of 90°, and data was collected with an integration time of 2 s to avoid saturation of the detector. A microspot with diameter of 50 μm was used. The %T values are obtained averaging values at wavelengths of 400 nm, 500 nm, 600 nm, 700 nm, 800 nm, and 900 nm and without subtracting the contribution of the substrate.

#### Electrical Characterization

Electrical resistivity of the LSCO thin film on LAO was determined using the Van der Pauw method over the 150–350 K temperature range with a Quantum Design Physical Property Measurement System (PPMS). For that purpose, Au electrodes of 1 mm × 1 mm were placed at the vertices of the films by means of the DC-sputtering system from TSST. Due to limitation of the PPMS instrument, the resistivity was measured only in the temperature range that provided resistances below ∼10^7^ Ω. The resistivity was obtained from sheet resistance and considering the film thickness.

#### Structural Characterization

The crystalline structure and phase purity of LSCO films were studied by X-ray diffraction (XRD) using a Bruker-AXS A25 D8 Discover instrument equipped with a Cu anode (Cu Kα, λ = 1.5418 Å). XRD θ–2θ scan measurements were performed in the range of 20–80°. The reciprocal space map (RSM) was acquired along the (103)-LSCO reflection. The in-plane and out-of-plane crystalline texture analysis was performed through phi and rocking curve scans along the (103) and (003) reflections of LSCO, respectively.

Aberration corrected scanning transmission electron microscopy (STEM) images and monochromated electron energy loss spectroscopy (EELS) spectra were acquired using a Nion HERMES-100 operated at 60 kV, at the University of Chinese Academy of Sciences, Beijing, China. Cross-sectional STEM specimens were prepared using the standard focused ion beam (FIB) lift-out process in a Thermo Fisher Scientific FIB system. The high-angle annular dark-field (HAADF) images were acquired using an annular detector with collection of semiangles of 75–210 mrad. The probe-forming semiangle was 32 mrad, and the collection semiangle for the EELS spectrometer was 75 mrad. After monochromation, the beam current was ∼20 pA and the best energy resolution obtained was ∼0.1 eV with the beam placed on the sample. To record the spectra, a dispersion of 0.05 eV per channel was used, and a set of 40 spectra with 500 ms time per acquisition were collected while scanning on the film to prevent radiation damage and subsequently aligned and integrated. The integrated spectra were slightly smoothed using a 3 channel low pass filter to reduce noise.

#### Surface Chemical Composition

X-ray photoelectron spectroscopy (XPS) measurements were performed with a SPECS PHOIBOS 150 hemispherical analyzer (SPECS GmbH, Berlin, Germany) using a monochromatic Al Kα radiation (1486.74 eV) source at 300 W at the Institut Català de Nanociencia i Nanotecnologia, Barcelona, Spain. The samples were analyzed with a spot size of 3.5 mm × 0.5 mm at a base pressure of 4 × 10^10^ mbar. Pass energies of 20 and 50 eV and step sizes of 0.05 and 1 eV were used for the high-resolution and survey spectra, respectively. The acquired spectra were processed with CasaXPS software using Shirley background subtraction. Binding energies were calibrated using C 1s at 284.9 eV.

## Results and Discussion

Among the wide variety of metal precursors available for solution processing, metal nitrates have proved superior to metalorganics in terms of requiring lower processing temperatures, and they generally produce denser films with fewer impurities.^39^ Indeed, preliminary studies conducted in our group using metalorganic precursors led to nonhomogeneous and unstable precursor solutions resulting in dramatically porous films; see Figure S1. Therefore, this study is focused on La, Sr, and Cr nitrates combined with different solvents (2-methoxyethanol, MOE, acetic acid, AcA, and water) and additives (*N*,*N*-dimethylformamide, DMF, acetylacetone, AA, diethanolamine, DEA, and citric acid, CA). According to their chemical formulations, the four solutions investigated are named MOE, DMF-AA, DEA, and CA; see Experimental Section and Table 1. The solutions prepared are homogeneous and free of precipitates, and the oxidation state of chromium gives rise to blueish/greenish coloration. The solution viscosity measured at room temperature increases from 0.0017 Pa·s for CA, 0.0043 Pa·s for MOE, and 0.0045 Pa·s for DMF-AA to 0.0085 Pa·s for DEA, in accordance with the corresponding molecular weight of the solvents and additives. Importantly, these values of viscosity ensure good wettability of the working single crystal perovskite substrates.^40^

To guarantee a controlled transformation process from the precursor solution to the formation of pure phase LSCO, simultaneous TGA-DSC analysis has been carried out from the dried gel of the four solutions described above. Details of the TGA-DSC conditions can be found in the experimental section. Note that the O_2_-rich atmosphere has been used to study the decomposition of all the solutions. Additionally, the decomposition of DEA solution has been also studied in air atmosphere because of the well-known violent and exothermic decomposition of diethanolamine in O_2_.^41^

**Figure 1:**
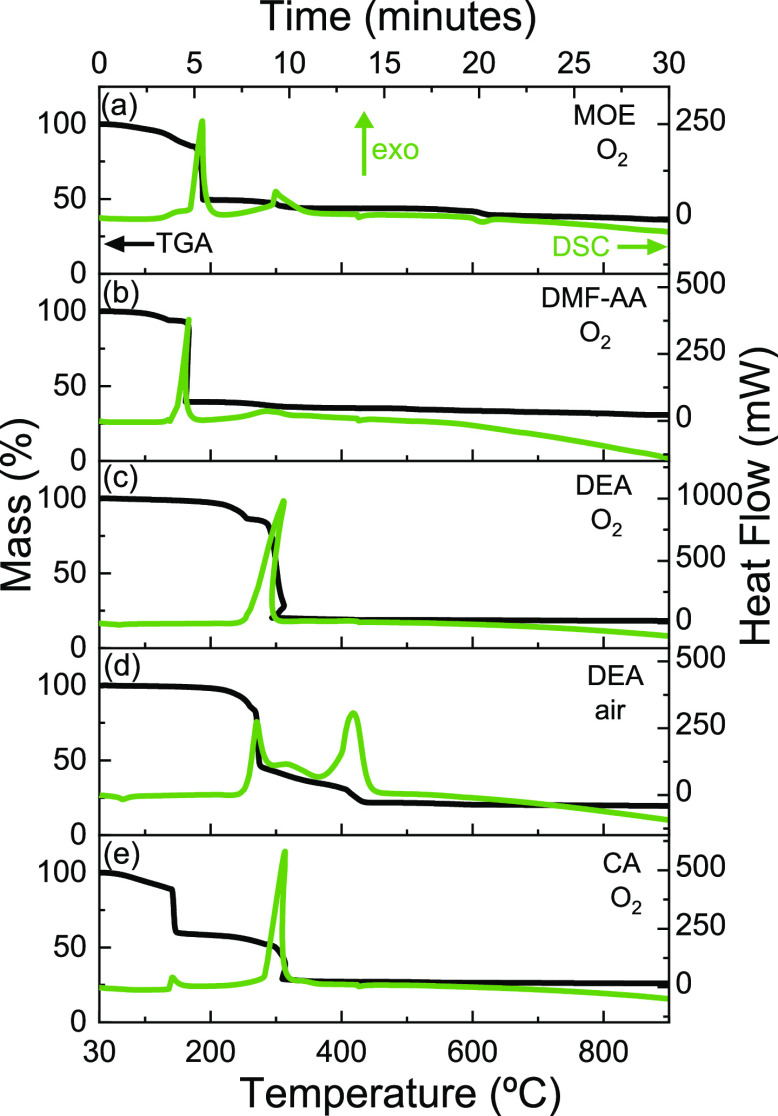
TGA-DSC curves obtained at 30 °C·min^–1^ from 30 to 900 °C in an O_2_ atmosphere for (a) MOE, (b) DMF-AA, (c) CA, and (d) DEA and (e) in air atmosphere for DEA. The positive and negative values of heat flow in the DSC curves are set to represent exothermic and endothermic phenomena, respectively.

The TGA curves of the dried gel of MOE, DMF-AA, CA, and DEA solutions in O_2_-rich atmosphere, Figure 1a–d, show the main mass losses at temperatures below 320 °C (>50%), which are related with two exothermic DSC peaks in MOE, DMF-AA, and CA and one exothermic peak in DEA. At these temperatures, according to the reported thermal decomposition of the individual metal nitrates,^39^ simultaneous phenomena occur including dehydration of metal nitrates to form concentrated salt solutions, the condensation of Cr(NO_3_)_3_, and the elimination of NO_*x*_ and CO_2_ as reaction product gases.^39,42^ The decomposition of the solvent 2-methoxyethanol into CO_2_ and CO^43^ is expected to occur in the same temperature range. Note that the use of additives in DMF-AA, DEA, and CA entails the formation of intermediates such as aconitic acid in CA^40,44^ and modifies the covalency of the metal–ligand bonds, changing the decomposition temperatures of the different thermal events when compared to pure nitrate decomposition.^30,39,43,45,46^ In addition, in the case of DEA, the strong exothermic DSC peak at 310 °C is related to the violent oxidative decomposition of diethanolamine to NH_3_ and acetic and formic acid.^41,47^ At temperatures above 320 °C, the decomposition of acetic acid into CH_4_ and CO_2_^48^ and the condensation of La(NO_3_)_3_ and Sr(NO_3_)_2_, which release gases such as H_2_O, NO_*x*_, and CO_2_, take place.^39^ These events are related to the presence of endothermic peaks at temperatures of ∼400 °C and ∼600 °C, respectively. Moreover, the downward trend of the DSC curve observed at high temperatures could indicate the presence of an endothermic peak above 900 °C (not shown here) that could be related to the crystallization of LSCO.^49,50^

The effect of the atmosphere on the thermal decomposition of the derived gel of DEA has been also evaluated. In air atmosphere, Figure 1e, the mass loss is more gradual than in oxygen, Figure 1d, and it has three exothermic peaks of less intensity compared to the one observed in oxygen, suggesting a milder decomposition.

Based on this study, fine-tuning of the thermal profile parameters (temperature, interim steps, annealing time, and heating ramps), Figure S2, has been performed to achieve a complete conversion of the precursor gel to pure-phase, epitaxial, and dense LSCO films. For the purpose of film processing optimization (details in Figure S3) and transmittance measurements, precursor solutions have been deposited on (00*l*)-STO single crystal substrates. It is anticipated that in order to be able to perform the electrical characterization of such films, LSCO has been also prepared on (00*l*)-LAO single crystal substrates, as described below.

**Figure 2:**
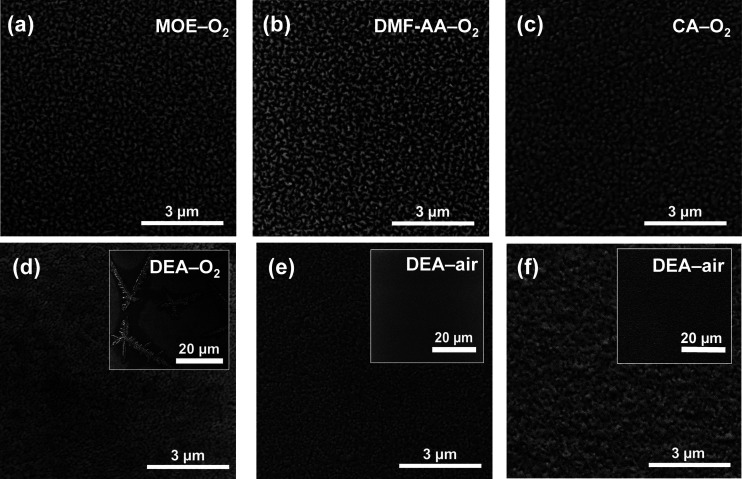
Scanning electron microscopy images of LSCO thin films on the STO substrate prepared from the (a) MOE–O_2_ atmosphere, (b) DMF-AA-O_2_ atmosphere, (c) CA–O_2_ atmosphere, (d) DEA–O_2_ atmosphere, (e) DEA–air atmosphere, and (f) LSCO on LAO substrate from DEA–air.

The effect of the solution chemistry on the surface morphology of LSCO thin films has been evaluated by SEM; see Figure 2. LSCO films obtained from MOE, DMF-AA, and CA solutions and processed in O_2_ atmosphere exhibit similar homogeneous and precipitate-free surface morphology, Figure 2a–c. However, they present rather unconnected LSCO grains that could impair the transport properties of the film, as it has been already reported for analogous (La,Sr)MnO_3_ films.^51^ LSCO films from DEA processed in O_2_ atmosphere present an enhanced grain connectivity that is probably attained by the chelating effect of diethanolamine, Figure 2d, but this solution chemistry favors the formation of large Cr-rich, star-shaped precipitates (inset) as confirmed by SEM-EDX in Figure S4. In contrast, when LSCO films from DEA solution are processed in air, Figure 2e, the formation of such precipitates is successfully avoided obtaining highly homogeneous films with dense surface morphology, suggesting that the Cr-rich precipitates present in Figure 2d could be formed during the strong exothermic decomposition reaction identified in the oxidizing atmosphere in the TGA-DSC analysis of Figure 1d. Note that *c*-axis growth has been obtained for all solution chemistries, Figure S5, whereas a wider θ–2θ scan confirmed phase pure and (00l) oriented growth of the films, Figure S6.

On the basis of these results, we have been able to establish an optimized process to prepare dense and epitaxial LSCO thin films on STO from DEA solution processed in air atmosphere, demonstrating the strong influence of solution chemistry and processing conditions on thin film surface morphology quality.

**Figure 3:**
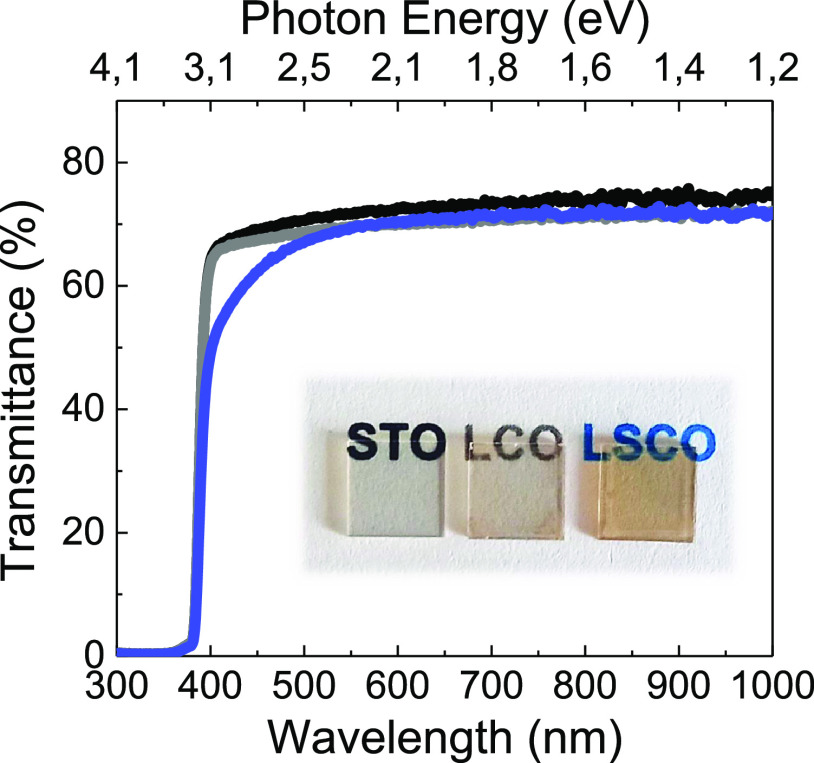
Optical transmittance spectra of STO substrate and 45 nm - LCO and 45 nm - LSCO thin films on STO from 300 to 1000 nm energy range. Inset shows photographs of the substrate and the samples.

The optical transmittance of ∼45 nm - LSCO films has been obtained with an spectroscopic ellipsometer in photometry mode from 300 to 1000 nm and compared to that of the transparent, although insulating, LCO parent compound film, Figure 3. As expected, the transmittance of LCO is very similar to that of the STO substrate, whereas the LSCO film presents absorption near 450 nm because of the slight brown coloration, as it can be observed in the pictures below the transmittance curve. The LSCO film presents an average optical transmittance of ∼67%, which is comparable to that of 67 nm - La_0.88_Sr_0.12_CrO_3_ on STO (%T = 63.4%) and to the *n*-type TCO counterparts 50 nm - SrVO_3_ and 40 nm - CaVO_3_, which reported %T values of ∼60%.^13,14^

**Figure 4:**
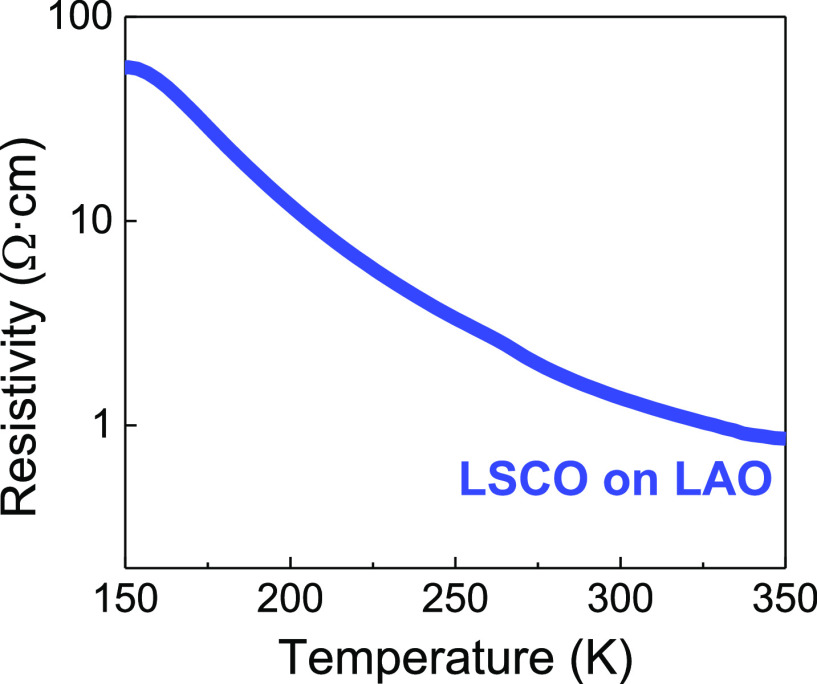
Temperature dependence of the electrical resistivity of 45 nm - LSCO on LAO from 150 to 350 K.

The electrical resistivity of vacuum deposited epitaxial LSCO films is reported to be higher on STO substrates than on LAO due to strain effects.^24−26^ Indeed, no measurable electrical conductivity was found for our CSD-LSCO films on STO (not shown). Therefore, for the purpose of this study, the electrical properties of ∼45 nm - LSCO films have been measured on LAO substrates. Note that these films present homogeneous, dense, and slightly rougher surface morphology than LSCO on STO probably due to the large lattice mismatch, see Figure 2f and Figure S8b. Figure 4 shows the temperature dependence of LSCO, ρ(T), measured in the range of 150–350 K. The resistivity at room temperature, 300 K, is 1.4 Ω·cm, one order of magnitude higher than that expected from LSCO films with the same Sr doping concentration on LAO substrates.^24^ This is also reflected on the residual resistivity ratio (RRR) calculated from ρ(300 K)/ρ(150 K) resulting in smaller values (0.024) than MBE films.^24^ The same trend has been reported for other CSD-processed *n*-type TCO perovskite thin films when compared to their vacuum-processed counterparts.^33,35,36,38,52,53^

**Figure 5:**
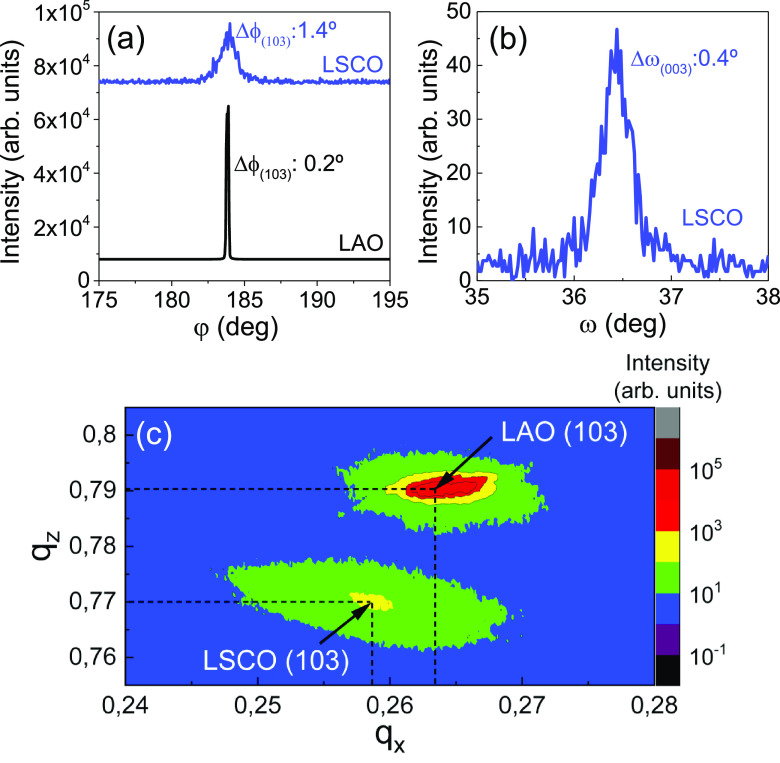
Structural study of the LSCO thin film grown on the LAO substrate. (a) Phi scan of LSCO compared to LAO, (b) rocking curve of LSCO, and (c) RSM around the (103) LSCO reflection.

In order to better understand the origin of this behavior, a detailed structural and strain analysis has been performed on LSCO films grown on LAO. In-plane texture of LSCO on LAO has been investigated from a phi-scan, Δϕ_(103),*LSCO*_ = 1.4° and Δϕ_(103),*LAO*_ = 0.2°, and the out-of-plane texture from the rocking curve, Δω_(003),*LSCO*_ = 0.4°, which confirm that films are biaxially textured; see Figures 5a,b. From the XRD-RSM, Figure 5c, the in-plane, *a*, and out-of-plane, *c*, lattice parameters of LSCO have been extracted considering a pseudocubic symmetry, resulting *a* = 3.867 ± 0.023 Å and *c* = 3.896 ± 0.005 Å (*a*_*LSCObulk*_ = 3.876 Å).^24−26^ The films show subtle in-plane compressive strain of −0.232% and an out-of-plane tensile strain of 0.516%. These results were further confirmed from strain maps by STEM images using geometrical phase analysis (GPA), Figure S7. This behavior differs from the results reported from MBE-LSCO films on LAO, which displayed −2.1% compressive strain.^24,26^ This difference could be attributed to the different nucleation and growth thermodynamics of CSD films,^54^ which tend to produce relaxed and epitaxial films with the formation of structural defects.^55−58^

**Figure 6:**
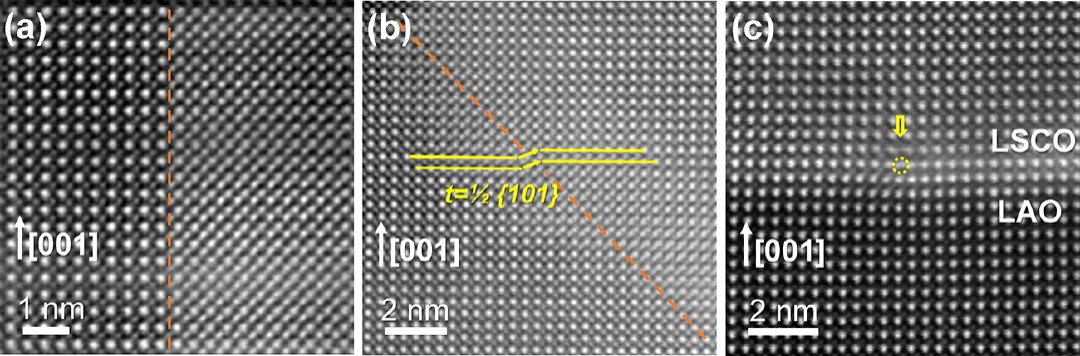
Structural defects found in LSCO thin films. (a, b) High-resolution HAADF images of antiphase boundaries where the boundary plane runs along the (010) and (011) crystallographic planes, respectively. (c) shows a dislocation core nucleated in the LSCO film near the substrate interface.

Deeper exploration of the crystalline structure of LSCO through high-resolution STEM HAADF images indeed reveals the formation of a significant amount of two types of structural defects in the film, namely, antiphase boundaries and misfit dislocations. Figure 6a,b shows two types of antiphase boundaries running along the (010) and (011) perovskite crystallographic planes, respectively. In these defects, one BO_2_ plane (from ABO_3_ structure) is suppressed, and the two crystals are relatively shifted along a glide plane with a ^1^/_2_{111} shift vector, forming a rock-salt structure at the fault. The glide plane will overlap the A and B sites of the perovskite structure when viewed perpendicular to the fault plane, as in the right area in Figure 6a; thus, the relative Z-contrast of the A and B site are equated. Figure 6b shows an edge-on image of the antiphase boundaries with a relative shift of ^1^/_2_{101} of the crystal structure at both sides of the antiphase boundary plane. Additionally, the formation of misfit dislocations is also observed near the substrate interface, Figure 6c. The misfit dislocations may form an array along the interface as observed in the GPA analysis in Figure S7. These extended defects are spontaneously formed to relax the misfit strain imposed by the substrate. Therefore, it is suggested that structural defects influence the resistivity behavior in our LSCO films on LAO substrates. Notoriously, the detrimental effect of structural defects such as antiphase boundaries on the electrical resistivity has already been reported in Fe_2_O_3_ films.^59^ Additionally, preliminary studies of the influence of LSCO film roughness and thickness on the ρ(T) behavior indicate that film resistivity increases in films with rougher surface morphology and poorly connected grains (Figure S8). Indeed, it has been widely studied that surface imperfections in transition metal complex oxides can produce significant changes on the electrical properties, as previously reported for other perovskite oxides (i.e., La_1–*x*_Sr_*x*_MnO_3_).^60,61^ Further analysis of ρ(T) indicates that the data can be fitted to both band conduction and polaron hopping transport models, suggesting a competition between these two mechanisms,^24^ and details on the fitting and activation energies can be found in Figure S9.

**Figure 7:**
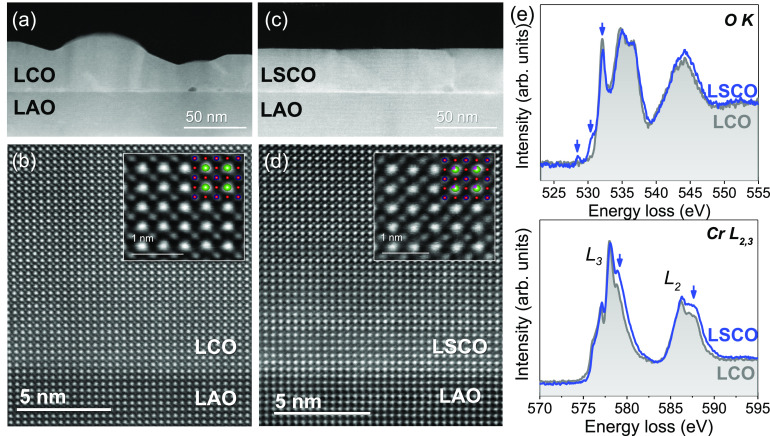
Z-contrast cross-section STEM images of (a, b) LCO on LAO and (c, d) LSCO on LAO. The insets in (b, d) are atomic-resolution STEM images of the LCO and LSCO structures with the atomic models overlaid, where green atoms correspond to La, red to Cr, and pink to Sr. (e) O K-edge and Cr L_2,3_-edge monochromated-EELS spectrum of LCO and LSCO on LAO. The blue arrows point to the characteristic spectral changes induced by the Sr doping.

To gain further understanding on the role of Sr doping on the crystallinity and Cr 3*d* - O 2*p* orbital hybridization in the CSD-LSCO films on LAO, cross-sectional STEM-EELS have been acquired and compared to LCO. Figure 7a,c shows continuous ∼45 nm - LCO and LSCO films, with clearly improved homogeneity and smoothness upon Sr-doping. Moreover, higher magnification STEM images, Figure 7b,d, confirm the epitaxial growth of LCO and LSCO films on the LAO substrate with atomically sharp interfaces. The higher resolution inset images show atomic resolution images of both LCO and LSCO structures viewed along the [100] direction.

Due to the low concentration of Sr-doping in the LSCO films, no obvious Z-contrast difference or structural changes are observed when compared to the undoped LCO. In order to investigate possible electronic structure changes as a consequence of Sr-doping, the O K-edge and Cr L_2,3_-edge spectra of CSD-LCO and LSCO have been acquired by means of monochromated-beam EELS; see Figure 7e. The spectral Cr L_2,3_-edge of LCO shows three main peaks between 573 and 583 eV, which correspond to the L_3_ line, and two peaks between 583 and 591 eV from the L_2_ line. The energies and line shapes of these peaks are characteristic of Cr^3+^, as it has been reported for Cr_2_O_3_ and LCO.^24,62^ On substituting La by Sr, it is observed that the Cr L_3,2_ line shape pattern changes, and the Cr L_3_ line-width broadens; the peaks indicated by blue arrows increase in intensity, turning toward spectral signatures representative of Cr^4+^. These features would be consistent with the localization of holes doped in a lattice of Cr ions.^24,63^

Moreover, the spectral O K-edge provides further details related with the unoccupied density of states of transition metal character. For LCO, a set of three peaks is observed between 525 and 539 eV, in good agreement with earlier reports of LCO films.^24^ Upon Sr-doping, two new features at 528.5 and 531 eV and the decrease in intensity of the peak at 532.5 eV are observed, indicated by the blue arrows in Figure 7e, characteristic of Sr-doped LCO and related to the appearance of new unoccupied states and partial hybridization between O 2*p* and Cr 3*d* orbitals from the bottom of the conduction band.^23,24^ These features are in line with the observed shift to lower binding energies of the XPS core-level spectra of Cr 2*p*, La 3*d*, and O 1*s* of LSCO films compared to LCO; see Figures S10 and S11, which can be associated with the modification of the chemical potential toward the valence band. This behavior appears to be a common phenomenon of hole-doped perovskite oxides^25,64,65^ and could explain the introduction of holes in our Sr-substituted LCO films and, therefore, the enhanced electrical conductivity compared to the highly resistive LCO.

## Conclusions

In this study we have developed a cost-efficient chemical route to prepare LSCO thin films. We demonstrate that the solution chemistry strongly influences the film morphology in which the combination of nitrate precursors, 2-methoxyethanol, acetic acid, and diethanolamine processed in air results in dense, epitaxial, and slightly strained films. These LSCO films display ∼67% optical transparency in the visible spectrum being as good as those reported for some of the most promising state-of-the-art TCOs. The values of the electrical resistivity are roughly higher than those expected based on the film chemical doping and strain state, and it is attributed to the presence of structural defects. It is also revealed that Sr doping in LaCrO_3_ changes the electronic structure; unoccupied states are formed in O 2*p* together with the formation of Cr^4+^ which determine its physical properties. We envisage several approaches to further improve the CSD-LSCO film performance including the growth on lattice matched substrates to modulate the strain, to reduce the presence of antiphase boundaries, and refine the La/Sr ratio to further boost the conductivity without diminishing the optical transmittance. It is expected that this work will stimulate further investigations on the use and improvement of LSCO as a *p*-type transparent conducting perovskite oxide and will motivate additional experimental efforts on solution processing as a cost-effective and feasible route to develop functional complex oxides that can be of use in many energy and electronic applications.

## Supporting Information Available

TGA-DSC, optical microscope image, and SEM of LSCO on STO from metalorganic route; detailed description of thermal profiles and fine-tuning crystallization processing conditions for LSCO on STO; structural and morphological analysis of the LSCO series on STO and LAO [XRD, AFM, SEM-EDX]; further analysis of ρ(T) of LSCO on LAO as a function of film thickness and roughness; STEM and GPA strain analysis of LSCO on LAO; and XPS study of LCO and LSCO on LAO. (PDF)
