# Correction to “Chemical Synthesis of La_0.75_Sr_0.25_CrO_3_ Thin Films for *p*-Type Transparent Conducting Electrodes”

**DOI:** 10.1021/acs.chemmater.3c02381

**Published:** 2023-10-11

**Authors:** Pamela Machado, Roger Guzmán, Ramon J. Morera, Jordi Alcalà, Anna Palau, Wu Zhou, Mariona Coll

The authors have identified two typos in the manuscript that are corrected below.

**Abstract**

Original text:

“Monochromated electron energy loss spectroscopy allowed changes in the electronic structure in LSCO films to be determined, revealing the creation of Cr^4+^ and unoccupied states at the O 2*p* upon Sr-doping.”

The O K-edge low energy peaks can be related to transitions from O 1s to unoccupied density of states with partial O 2p character hybridized with Cr 3d states, as properly stated at different locations along the Discussion section. Therefore, in the above text O 2*p* was mistyped instead of O K-edge.

New text:

*Monochromated electron energy loss spectroscopy allowed changes in the electronic structure in LSCO films to be determined, revealing the creation of Cr*^*4+*^*and unoccupied states at the O K-edge upon Sr doping.*

**Conclusions**

Original text:

“It is also revealed that Sr doping in LaCrO_3_ changes the electronic structure; unoccupied states are formed in O 2*p* together with the formation of Cr^4+^ which determine its physical properties.”

The same typo occurs here, “O 2*p*” should read “O K-edge”.

New text:

*It is also revealed that Sr doping in LaCrO*_3_*changes the electronic structure; unoccupied states are identified at the O K-edge together with the formation of Cr*^*4+*^, *which determine its physical properties*.

**Discussion**

Original text:

“Upon Sr-doping, two new features at 528.5 and 531 eV and the decrease in intensity of the peak at 532.5 eV are observed, indicated by the blue arrows in Figure 7e, characteristic of Sr-doped LCO and related to the appearance of new unoccupied states and partial hybridization between O 2*p* and Cr 3*d* orbitals from the bottom of the conduction band.”

The sentence is incomplete and should read as follows.

New text:

*Upon Sr doping, two new features at 528.5 and 531 eV and the decrease in intensity of the peak at 532.5 eV are observed, indicated by the blue arrows in Figure 7e, characteristic of Sr-doped LCO and related to the appearance of new unoccupied states and partial hybridization between O 2p and Cr 3d orbitals. This band falls at lower binding energy than the bottom of the conduction band.*

Previous studies on valence band XPS and O K-edge XAS performed on La_1–*x*_Sr_*x*_CrO_3_ thin films, which are properly referenced in the text, revealed a transfer of spectral weight from the occupied Cr 3*d* t_2g_ valence band, suggesting a split-off unoccupied state (empty Cr 3*d*-derived band with partial O 2*p* character) above the Fermi level, resulting from the hole doping.

