# Supplementary material for: Chemical Synthesis of La0.75Sr0.25CrO3 Thin Films for p-Type Transparent Conducting Electrodes
Source: Chem Mater. 2023 Apr 13;35(9):3513–21. doi: 10.1021/acs.chemmater.2c03831 (PMC10173867; doi:10.1021/acs.chemmater.2c03831)
Supplement: Supplementary file 1 — cm2c03831_si_001.pdf [file cm2c03831_si_001.pdf]

Supporting Information for On the chemical synthesis of $La_{0.75}Sr_{0.25}CrO_3$ thin films for *p*-type transparent conducting electrodes

Pamela Machado,† Roger Guzmán,‡ Ramon J. Morera,† Jordi Alcalà,† Anna Palau,† Wu Zhou,‡ and Mariona Coll*,†

†*Institut de Ciència de Materials de Barcelona ICMAB-CSIC, Campus UAB, Bellaterra 08193, Spain*

‡*School of Physical Sciences, University of Chinese Academy of Sciences, Beijing 100049, China*

E-mail: mcoll@icmab.es

Preliminary study on metalorganic route for the preparation of LSCO thin films.

Preliminary studies have been conducted to evaluate metalorganic versus metal nitrate precursors for the preparation of LSCO thin films. For metalorganic precursors route: La, Sr, Cr acetates and a solvent blend of acetic acid and water (5:1) were initially selected but it led to non-homogeneous solutions with Cr-rich precipitates (not shown here). As an alternative, Cr acetylacetonate was tested in combination with La and Sr acetates and using the same solvent blend (acetates-acetylacetonate route), that resulted in an homogeneous solution.

TGA/DSC analysis allowed to identify the starting conditions for the thermal treatment to stabilize the LSCO phase, see Figure S1(a). The TGA curve shows the main mass loss at temperatures below 290 °C (~50%). At these temperatures, simultaneous phenomena occur, including dehydration of metal precursors and decomposition of Cr acetylacetonate into acetone and CO_2, which is related the endothermic peak at ~210 °C.[1,2] At temperatures above 300 °C decomposition of acetic acid into CH_4 and CO_2,[3] and La and Sr metal precursors takes place. La acetate decomposes into carbonates (300 - 350 °C) and oxycarbonates (~450 °C)[4] and Sr acetate into carbonates (~450 °C).[5] In addition, the reaction product gases and acetone decompose at temperatures above 500 °C.[6] These events are related to the presence of exothermic peaks at temperatures of ~430 °C and ~570 °C. The downward trend of DSC curve observed at high temperatures could indicate the presence of an endothermic peak above 900 °C that could be related with the crystallization of LSCO (not shown here).[7,8] Before deposition, rheometry study of the solution revealed a viscosity of 0.0042 Pa·s, which has been reported to ensure effective wettability of single crystal perovskite substrates.[9] However, our spin coated films resulted with inhomogeneities unraveling solution instability, Figure S1(b), that ultimately resulted in a dramatically porous film, Figure S1(c). Envisaging a very challenging process to improve the quality of the film, it has been decided to switch to the use of nitrate precursors.

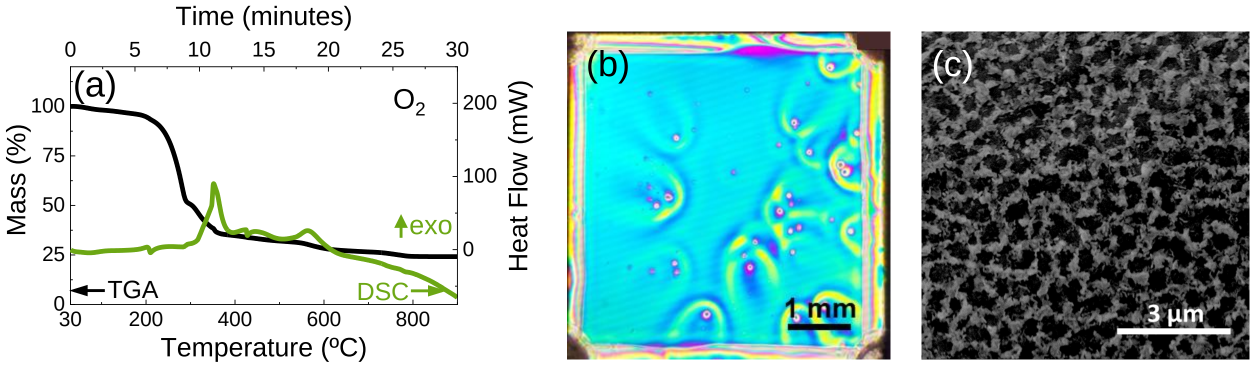

Figure S1: Evaluation of the preparation of LSCO thin films from acetates-acetylacetonate route. (a) TGA-DSC curves obtained at 30 °C·min^{-1} from 30 °C to 900 °C in O_2 atmosphere. (b) Optical microscope image of the as-deposited film at 6000 rpm for 30 seconds and (c) SEM image of the resulting LSCO thin film grown on (00l)-STO at 850 °C for 45 minutes under O_2 flow.

Thermal profile and fine tuning of processing conditions for the preparation of LSCO thin films from metal nitrate precursors.

The best conditions for crystallization of LSCO thin films on (00l)-STO substrate have been determined through fine tuning of the temperature (700 - 900 °C) and dwell time (15 - 45 min) according of the TGA-DSC analysis. Figure S2 shows the optimized thermal profile to prepare LSCO thin films from the four precursor solutions here studied, MOE, DMF, CA and DEA. As a case example, the influence of the processing conditions on the surface morphology and crystallinity has been evaluated for the MOE solution by XRD θ-2θ scan and SEM, Figure S3. Based on this study, the optimized crystallization processing involves heating the samples up to 850 °C for 45 minutes with heating and cooling ramps of 660 °C/minute.

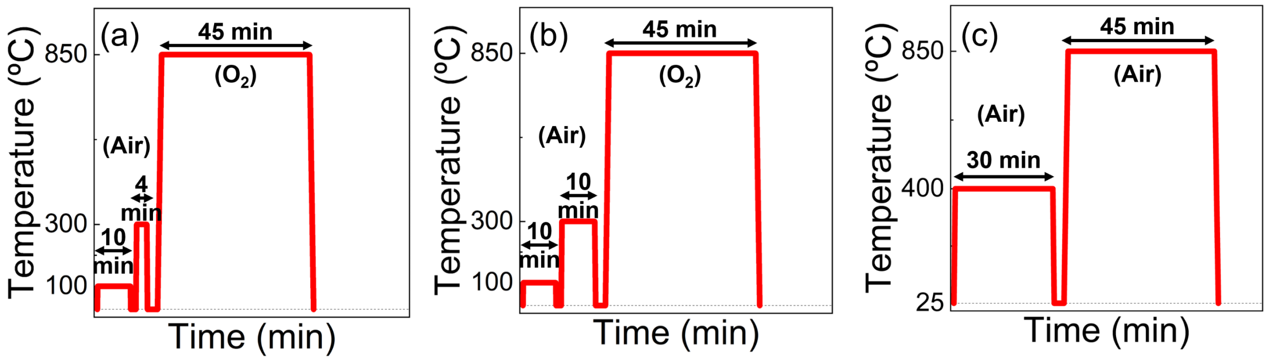

Figure S2: Thermal profiles defined for the preparation of LSCO films from (a) MOE, DMF-AA and CA, (b) DEA in O_2 and (c) DEA in air.

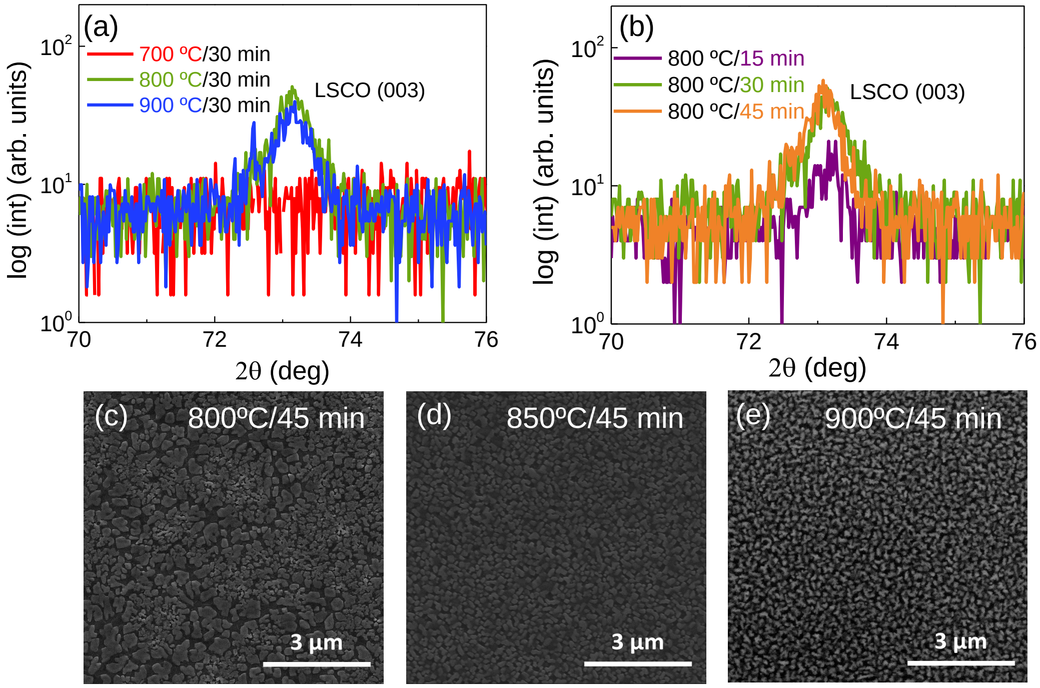

Figure S3: XRD θ-2θ scans of LSCO thin films grown on (00l)-STO from MOE solution under different (a) annealing temperatures and (b) annealing times. SEM images for films grown at (c) 800 °C, (d) 850 °C and (e) 900 °C for 45 minutes.

SEM-EDX analysis have been performed in LSCO films from DEA solution processed in O_2 atmosphere to identify the nature of star-shaped precipitates. Figure S4(a) shows the SEM image of the studied zone of the sample, indicating the two areas that are analyzed by

EDX. Area 1 corresponds to the homogeneous matrix of the film and area 2 to the precipitate. From the EDX analysis, Figure S4(b), C, O, Sr, La, Ti and Cr have been identified. The area corresponding to the precipitate presents higher content of Cr than that of the homogeneous zone of the film, indicating the Cr-rich nature of the star-shaped precipitate.

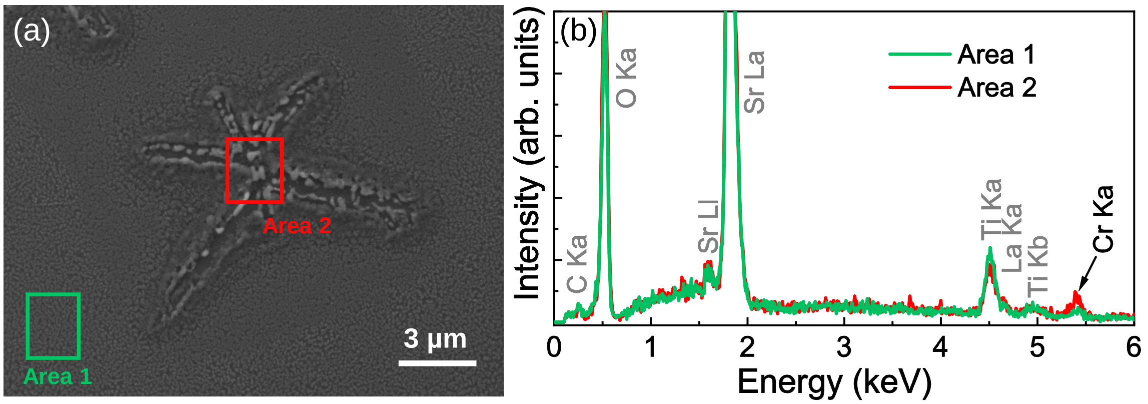

Figure S4: SEM-EDX analysis of LSCO thin film from DEA solution grown on (00l)-STO under O_2 atmosphere. Note that SEM image shows the analyzed areas for EDX spectra.

Then, the influence of the precursor chemistry on film crystallinity has been evaluated. Figure S5 shows the XRD θ-2θ scan of LSCO thin films prepared on (00l)-STO substrates from the different solution chemistries studied in this work. Films obtained from MOE, DMF-AA, CA have been prepared under O_2 atmosphere and LSCO from DEA under both O_2 and air atmospheres. The main Bragg reflection at 72.55° corresponds to (003) STO substrate whereas the reflection at ∼73.15° can be assigned to (003) LSCO, evidencing a c-axis oriented growth for all solution chemistries. Note that subtle variations in (003) LSCO Bragg reflection intensity can be observed, mostly attributed to the slight difference in film thickness, 40 nm for MOE films vs 70 nm for CA films.

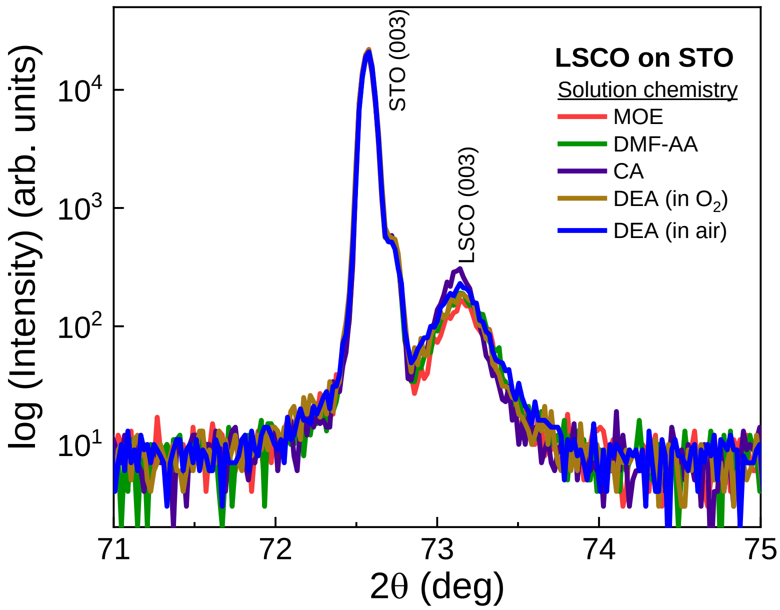

Figure S5: XRD θ-2θ scan of LSCO thin films from different solution chemistries grown on (00l)-STO substrate.

Additional θ-2θ XRD scans have been performed on LSCO from DEA processed in air atmosphere. Figure S6(a,b) reveal the presence of (00l) reflections corresponding to LSCO and STO or LAO substrates, respectively, confirming phase pure and (00l) oriented growth of the films.

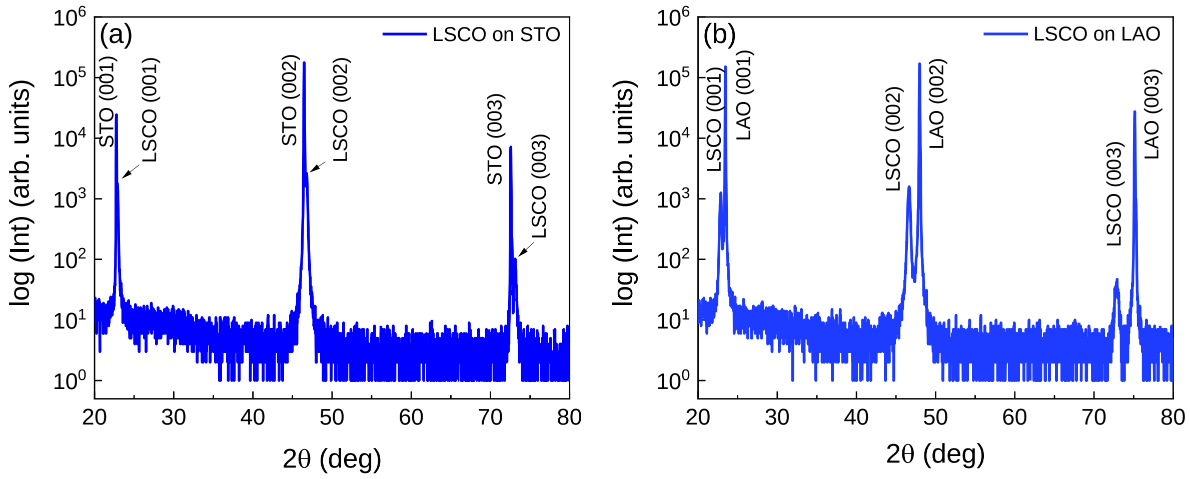

Figure S6: XRD θ-2θ scan of LSCO from DEA-air on (a) (00l)-STO and (b) (00l)-LAO.

Structural analysis

To gain more insights in the local strain state of the LSCO films, we analyzed high-resolution STEM images with the geometrical phase analysis (GPA) tool. Figure S7(a) is the HAADF images of LSCO films on LAO, seen along the [010] direction. The inset corresponds to the Fast Fourier Transform (FFT), where it is shown the splitting of the LSCO reflections from the ones of LAO, suggesting that the films are in a relaxed strain state. Masking out the g(100) and the g(001) reflections of the FFT, we computed the in-plane ϵ_{xx} and out-of-plane ϵ_{yy} mismatch strain component maps with compared to LAO substrate, being ∼+2.4 %, Figure S7(b,c). The color scale in the maps refers to the lattice spacing variation from a reference LAO substrate (a_{bulk} = 3.796 Å). From the maps, we also obtain a-LSCO ∼3.877 Å and c-LSCO ∼3.884 Å. These results reinforce the relaxed state of the film and they are in well agreement with the XRD measurements.

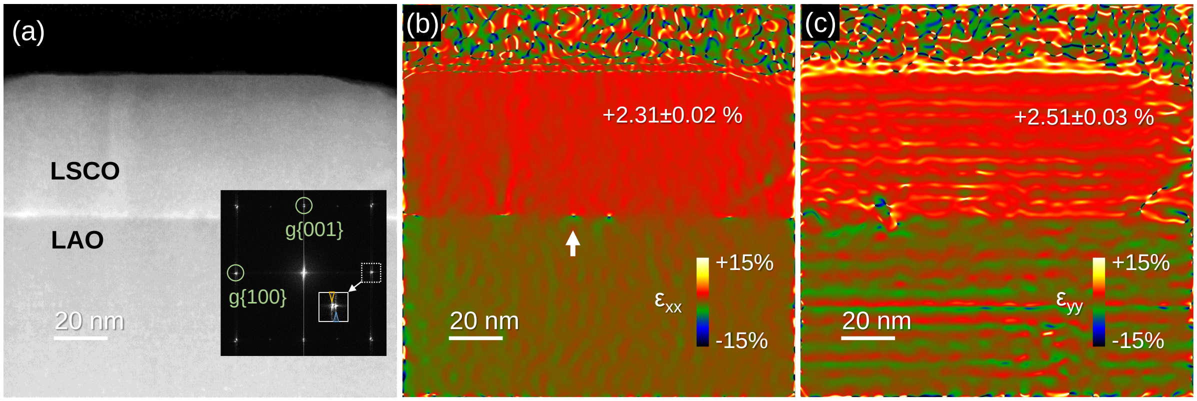

Figure S7: Geometrical phase analysis of the LSCO films. (a) HAADF cross sectional image of LSCO films grown on LAO substrate. The inset corresponds to FFT, where the yellow and blue arrows point to the splitting of the (100)-LSCO and (100)-LAO reflections, respectively. (b) In-plane ϵ_{xx} and (c) out-of-plane ϵ_{yy} strain component maps of the image in (a). The white arrow in the ϵ_{xx} map points to the formation of misfit dislocations at the interface.

Electrical characterization: influence of LSCO film thickness and fits of electrical resistivity to transport models.

The influence of LSCO film surface roughness and thickness on the electric resistivity, ρ, has been systematically evaluated in Figure S8. For this study, a series of LSCO films with different thickness have been prepared on (00l)-LAO substrate by modifying the concentration of the DEA precursor solution which is well known to be proportional to the thickness.[10] Precursor solutions of 0.12, 0.25 and 0.50 M have been prepared to fabricate LSCO films with theoretical thickness of 20 nm, 45 nm and 90 nm, respectively. The surface morphology of the corresponding films has been studied by AFM topography images, Figures S8(a-c). The thinnest film shows an inhomogeneous surface dictated by large pores and few outcrops. By increasing the film thickness the surface becomes more homogeneous with well defined grain size, typical of the CSD island-growth. The root mean square (rms) surface roughness calculated from these topographic images increases from 11 nm to 37 nm by increasing the film thickness, thus revealing a high surface roughness. The fact that the surface roughness

increases with thickness could be attained to the high concentration of solute in the precursor solution which has a direct impact on the growth kinetics, as previously reported in CSD-ZnO films.[11] On the other hand, XRD θ-2θ scans are depicted in Figure S8(d) and indicate c-axis orientation in all LSCO films. We also identified a gradual shift in the 2θ towards higher angles which could indicate film relaxation from epitaxial strain, Figure S8(d). Clear changes are observed in the intensity of (003) LSCO reflection that increases with solution concentration and can be related with film thickness and crystallinity.[12] The thickness of 0.25 M precursor solution is accurately determined from the TEM analysis, 45 nm (Figure 7 of main manuscript). Taking this thickness as a reference and correlated with the integrated (003) LSCO reflection intensity from XRD, it has been estimated that 0.12 M and 0.5 M solution produce films of $\sim$ 18 nm and $\sim$ 94 nm thickness, respectively. The ρ(T) has been measured and compared for the three films showing that the resistivity for the 18 nm and 94 nm films is notably higher at room temperature. The poor conductivity of these 18 and 94 nm films is further disclosed for the impossibility to acquire the ρ(T) for the whole range of temperatures (150 K - 350 K), Figure S8(e). These results evidence that the films that show a surface morphology with poorly connected grains lead to high resistivity and it is very likely that this parameter dominates over the possible existence of epitaxial strain. Indeed, it is widely studied that surface imperfections in transition metal oxide films can widely produce changes on the electrical properties, as previously reported for other perovskite oxides (i.e. $La_{1\text{-}x}Sr_xMnO_3$).[13,14]

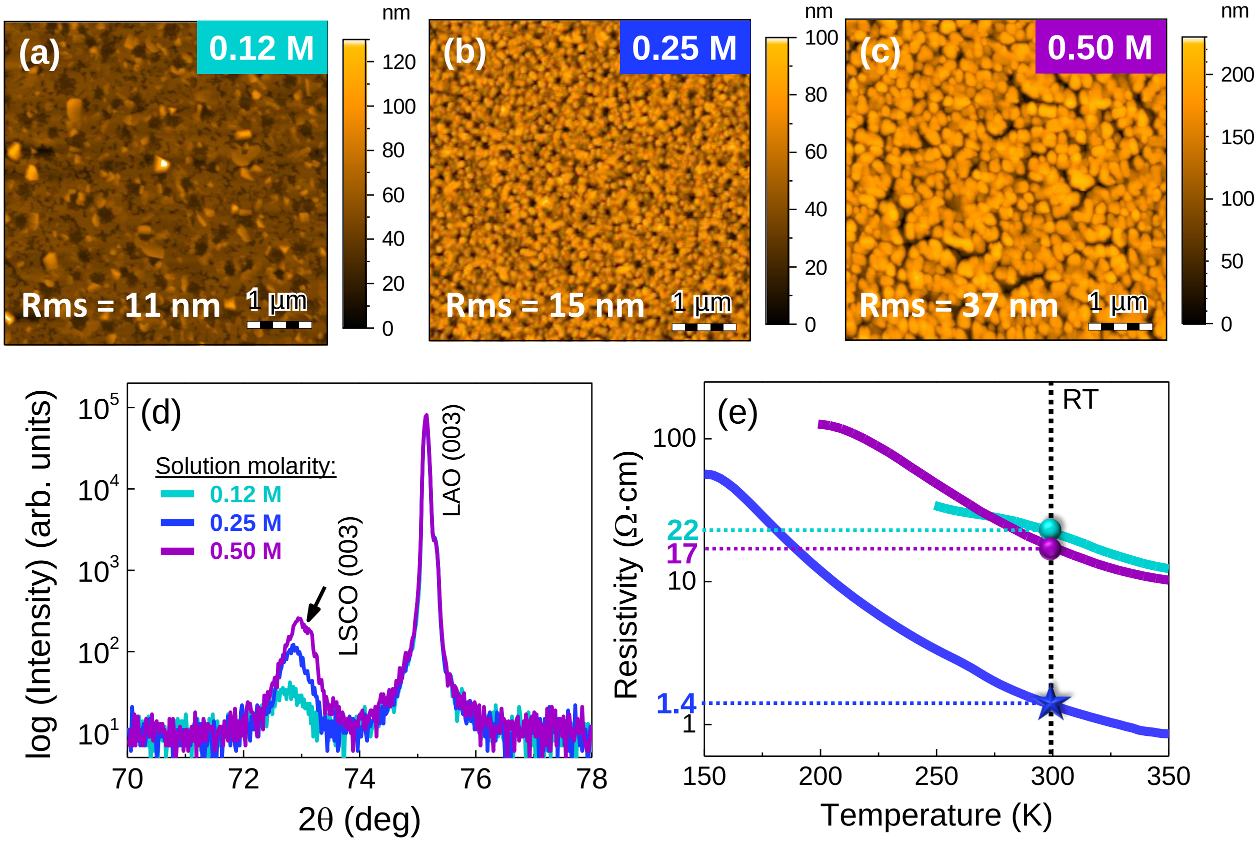

Figure S8: Systematic study on thickness-roughness influence on electric resistivity of LSCO on (001)-LAO from DEA formulation of 0.12 M ($\sim$ 18 nm), 0.25 M (45 nm) and 0.5 M ($\sim$ 94 nm). (a-c) AFM topography images (d) XRD θ-2θ scan and (e) temperature dependence of the electrical resistivity of the corresponding films.

Fits of resistivity measurements from 150 to 300 K to band conduction ($\rho \propto \exp(E_a/kT)$) and small polaron hopping ($\rho \propto \mathrm{T} \cdot \exp(\mathrm{E}_a/kT)$) transport models. The estimated activation energies (E_a) have been deduced from these fits and are 0.032 eV for band conduction model and 0.038 eV for polaron hopping model, lower than those reported for epitaxial MBE-LSCO thin films on (001)-LAO.[15]

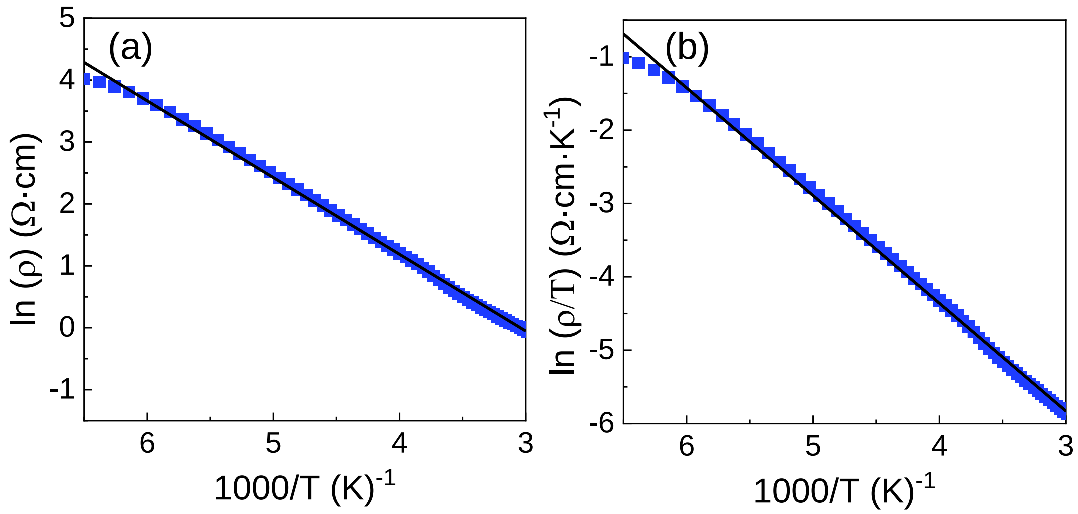

Figure S9: Fits of resistivity data to (a) band conduction and (b) polaron hopping transport models for LSCO film on LAO.

Chemical analysis of LSCO thin films by X-ray Photoelectron Spectroscopy.

In order to obtain further information on the surface chemical composition of undoped LCO and LSCO films, X-ray Photoelectron Spectroscopy (XPS) analysis has been performed. Figure S10 shows the core-level spectra of Cr *2p*, O *1s*, La *3d* and Sr *3d* calibrated in energy using the C *1s* centered at 284.9 eV, Figure S11.

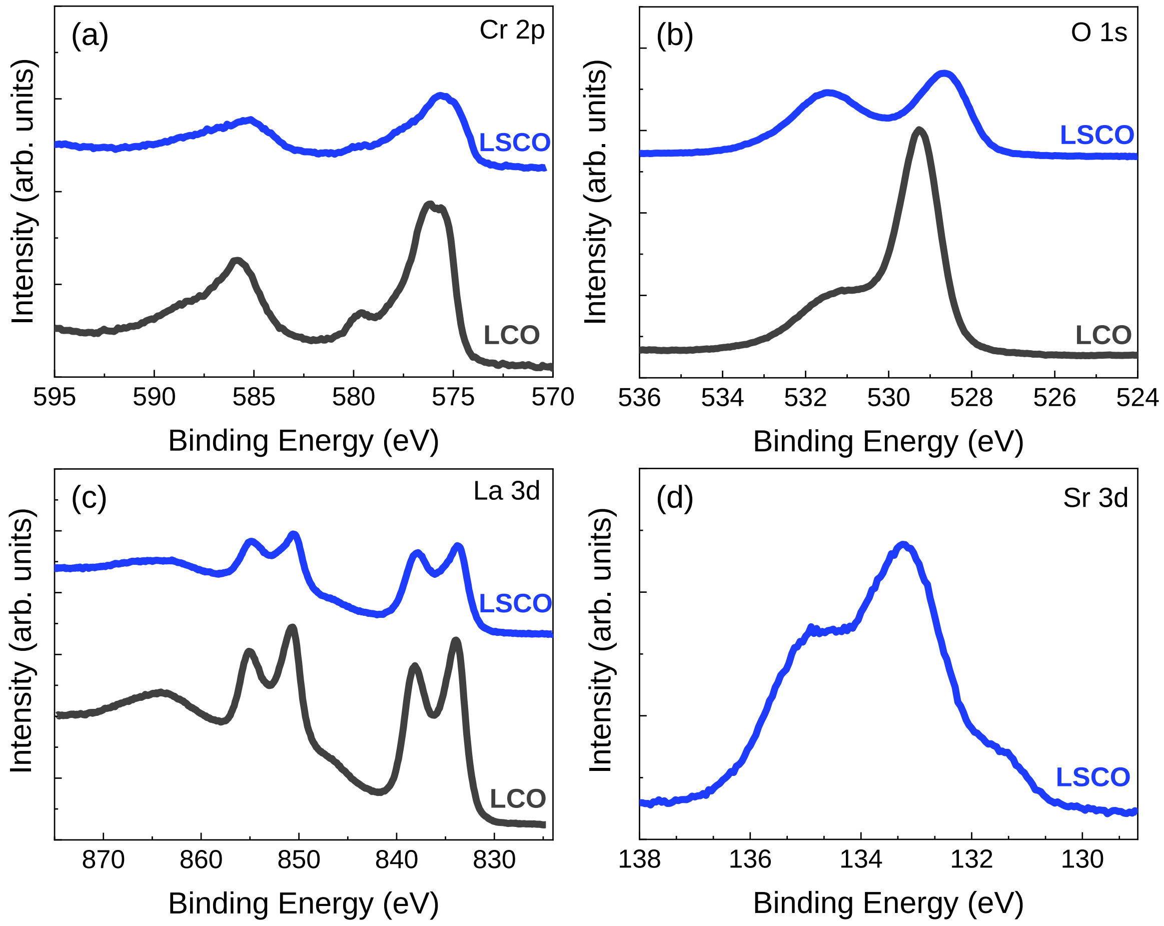

Figure S10: High resolution core level XPS spectra of (a) Cr *2p*, (b) O *1s*, (c) La *3d* and (d) Sr *3d* for LCO (grey) and LSCO (blue).

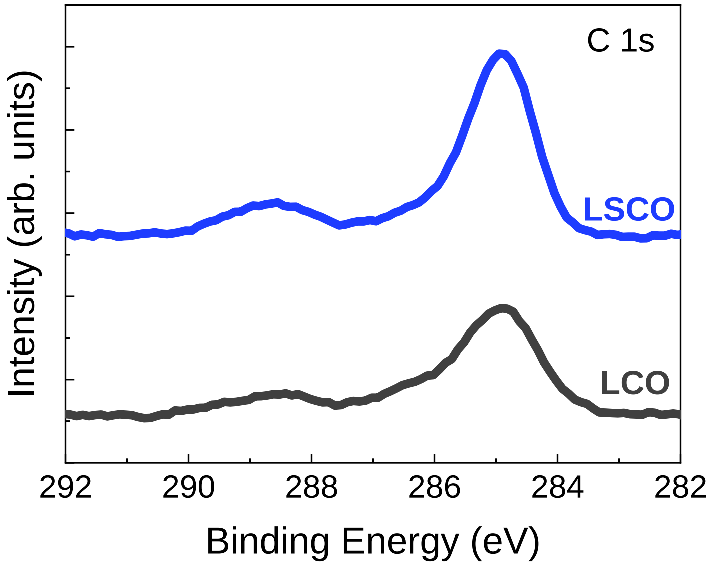

Figure S11: C *1s* core level spectra for LSCO and LCO films calibrated at 284.9 eV

The Cr *2p* spectra mainly show the fingerprint of Cr^{3+} species with Cr $2p_{3/2}$ peak centered at 576 eV for LCO and 575.5 eV for LSCO.[15–18] Both spectra also show a small contribution of Cr^{6+}, weak peak centered at ~579 eV, attributed to sample surface oxidation due to air exposure prior to XPS analysis.[15] Note that the presence of the Cr^{6+} hinders the unambiguous detection of the possible formation of Cr^{4+} species upon Sr-doping. The O *1s* core level spectra, Figure S10(b), show two contributions, the low-binding energy peak centered at 529.2 eV for LCO and 528.7 eV for LSCO, which corresponds to O-lattice in oxides, and the high-binding energy peak associated to adsorbed H_2O, OH and organic contamination.[15,19] The La *3d* spectra, Figure S10(c), show the typical La $3d_{3/2}$ and La $3d_{5/2}$ doublet peaks with a spin-orbital splitting energy of ~16.8 eV, characteristic of La^{3+}.[19] In this case, peak binding energies are downward shifted 0.3 eV for LSCO samples. Finally, the Sr *3d* core level spectrum, Figure S10(d), presents two peaks, at 134.9 and 133.2 eV, which are attributed to Sr $3d_{3/2}$ and Sr $3d_{5/2}$ and corresponds to Sr-O bonds. The shoulder at 132 eV could be attributed to Sr atoms with different chemical environment, already identified in other Sr-perovskite oxides.[20–22]

Notoriously, the line shapes of Cr *2p*, O *1s* and La *3d* are not affected by Sr-doping but the core level spectra show a nearly constant (0.2 - 0.3 eV) down shift in binding energy compared to LCO. This shift could be assigned to a change in chemical potential and appears to be a common phenomenon in other hole-doped perovskite oxides including (La,Sr)TiO_3 and (La,Sr)MnO_3.[15,23–25]

References

(1) Hoene, J. V.; Charles, R. G.; Hickam, W. M. Thermal Decomposition of Metal Acetylacetonates: Mass Spectrometer Studies. *The Journal of Physical Chemistry* **1958**, *62*, 1098–1101.

(2) Lalancette, R. A.; Syzdek, D.; Grebowicz, J.; Arslan, E.; Bernal, I. The thermal decomposition and analyses of metal tris-acetylacetonates. *Journal of Thermal Analysis and Calorimetry* **2019**, *135*, 3463–3470.

(3) Blake, P. G.; Jackson, G. E. The thermal decomposition of acetic acid. *J. Chem. Soc. B* **1968**, 1153–1155.

(4) Patil, K. C.; Chandrashekhar, G. V.; George, M. V.; Rao, C. N. R. Infrared spectra and thermal decompositions of metal acetates and dicarboxylates. *Canadian Journal of Chemistry* **1968**, *46*, 257–265.

(5) Duan, Y.; Li, J.; Yang, X.; Cao, X. M.; Hu, L.; Wang, Z. Y.; Liu, Y. W.; Wang, C. X. Thermal investigation of strontium acetate hemihydrate in nitrogen gas. *Journal of Thermal Analysis and Calorimetry* **2008**, *94*, 169–174.

(6) Davoud, J. G.; Hinshellwood, C. N. Thermal Decomposition of Acetone. *Nature* **1939**, *144*, 909–910.

(7) Ding, X.; Liu, Y.; Gao, L.; Guo, L. Synthesis and characterization of doped $LaCrO_3$ perovskite prepared by EDTA–citrate complexing method. *Journal of Alloys and Compounds* **2008**, *458*, 346–350.

(8) Liu, D.; Shi, P.; Liu, Y.; Zhang, Y.; Tian, B.; Ren, W. Optimizing the Properties of $La_{0.8}Sr_{0.2}CrO_3$ Thin Films through Post-Annealing for High-Temperature Sensing. *Nanomaterials* **2021**, *11*, 1–11.

(9) Salles, P.; Caño, I.; Guzman, R.; Dore, C.; Mihi, A.; Zhou, W.; Coll, M. Facile Chemical Route to Prepare Water Soluble Epitaxial $Sr_3Al_2O_6$ Sacrificial Layers for Free-Standing Oxides. *Advanced Materials Interfaces* **2021**, *8*, 2001643 (1–7).

(10) Schubert, D. W.; Dunkel, T. Spin coating from a molecular point of view: its concentration regimes, influence of molar mass and distribution. *Materials Research Innovations* **2003**, *7*, 314–321.

(11) Kamaruddin, S. A.; Chan, K.-Y.; Yow, H.-K.; Zainizan Sahdan, M.; Saim, H.; Knipp, D. Zinc oxide films prepared by sol–gel spin coating technique. *Applied Physics A* **2011**, *104*, 263–268.

(12) Liang, Y.-C.; Liang, Y.-C. Strain-dependent surface evolution and magneto-transport properties of $La_{0.7}Sr_{0.3}MnO_3$ epilayers on $SrTiO_3$ substrates. *Journal of Crystal Growth* **2007**, *304*, 275–280.

(13) Moreno, C. New features in solution derived $La_{0.7}Sr_{0.3}MnO_3$ thin films: spontaneous outcropping and nanoscale reversible resistive switching. Ph.D. thesis, Universitat Autonoma de Barcelona, 2010.

(14) Liao, Z.; Zhang, J. Metal-to-Insulator Transition in Ultrathin Manganite Heterostructures. *Applied Sciences* **2019**, *9*, 1–20.

(15) Zhang, K. H. L.; Du, Y.; Sushko, P. V.; Bowden, M. E.; Shutthanandan, V.; Sallis, S.; Piper, L. F. J.; Chambers, S. A. Hole-induced insulator-to-metal transition in $La_{1-x}Sr_xCrO_3$ epitaxial films. *Phys. Rev. B* **2015**, *91*, 155129.

(16) Chambers, S. A.; Droubay, T. Role of oxide ionicity in electronic screening at oxide/metal interfaces. *Phys. Rev. B* **2001**, *64*, 075410.

(17) Qiao, L.; Zhang, K. H. L.; Bowden, M. E.; Varga, T.; Shutthanandan, V.; Colby, R.; Du, Y.; Kabius, B.; Sushko, P. V.; Biegalski, M. D.; Chambers, S. A. The Impacts of Cation Stoichiometry and Substrate Surface Quality on Nucleation, Structure, Defect Formation, and Intermixing in Complex Oxide Heteroepitaxy–$LaCrO_3$ on $SrTiO_3$(001). *Advanced Functional Materials* **2013**, *23*, 2953–2963.

(18) Zhang, K. H. L.; Sushko, P. V.; Colby, R.; Du, Y.; Bowden, M. E.; Chambers, S. A. Reversible nano-structuring of $SrCrO_{3-d}$ through oxidation and reduction at low temperature. *Nature Communications* **2014**, *5*, 4669.

(19) Moulder, J.; Chastain, J. *Handbook of X-ray Photoelectron Spectroscopy: A Reference Book of Standard Spectra for Identification and Interpretation of XPS Data*; Physical Electronics Division, Perkin-Elmer Corporation, 1992.

(20) Wang, X.; Huang, K.; Yuan, L.; Xi, S.; Yan, W.; Geng, Z.; Cong, Y.; Sun, Y.; Tan, H.; Wu, X.; Li, L.; Feng, S. Activation of Surface Oxygen Sites in a Cobalt-Based Perovskite Model Catalyst for CO Oxidation. *The Journal of Physical Chemistry Letters* **2018**, *9*, 4146–4154, PMID: 29966086.

(21) Opitz, A. K.; Rameshan, C.; Kubicek, M.; Rupp, G. M.; Nenning, A.; Götsch, T.; Blume, R.; Hävecker, M.; Knop-Gericke, A.; Rupprechter, G.; Klötzer, B.; Fleig, J. The Chemical Evolution of the $La_{0.6}Sr_{0.4}CoO_{3d}$ Surface Under SOFC Operating Conditions and Its Implications for Electrochemical Oxygen Exchange Activity. *Topics in Catalysis* **2018**, *61*, 2129–2141.

(22) Feng, Z.; Crumlin, E. J.; Hong, W. T.; Lee, D.; Mutoro, E.; Biegalski, M. D.; Zhou, H.; Bluhm, H.; Christen, H. M.; Shao-Horn, Y. In Situ Studies of the Temperature-Dependent Surface Structure and Chemistry of Single-Crystalline (001)-Oriented $La_{0.8}Sr_{0.2}CoO_{3d}$ Perovskite Thin Films. *The Journal of Physical Chemistry Letters* **2013**, *4*, 1512–1518, PMID: 26282307.

(23) Fujimori, A.; Ino, A.; Matsuno, J.; Yoshida, T.; Tanaka, K.; Mizokawa, T. Core-level photoemission measurements of the chemical potential shift as a probe of correlated electron systems. *Journal of Electron Spectroscopy and Related Phenomena* **2002**, *124*, 127–138, Frontiers in photoemission spectroscopy of solids and surfaces.

(24) Horiba, K.; Chikamatsu, A.; Kumigashira, H.; Oshima, M.; Nakagawa, N.; Lippmaa, M.; Ono, K.; Kawasaki, M.; Koinuma, H. In vacuo photoemission study of atomically controlled $La_{1-x}Sr_xMnO_3$ thin films: Composition dependence of the electronic structure. *Phys. Rev. B* **2005**, *71*, 155420.

(25) Bein, N. S.; Machado, P.; Coll, M.; Chen, F.; Makarovic, M.; Rojac, T.; Klein, A. Electrochemical Reduction of Undoped and Cobalt-Doped $BiFeO_3$ Induced by Water Exposure: Quantitative Determination of Reduction Potentials and Defect Energy Levels Using Photoelectron Spectroscopy. *The Journal of Physical Chemistry Letters* **2019**, *10*, 7071–7076.
